# Comparative analysis of mycorrhizal communities associated with *Struthiopteris spicant* (L.) Weiss across Europe and North America

**DOI:** 10.3389/fpls.2024.1402946

**Published:** 2024-06-04

**Authors:** Thais Guillen-Otero, Dietrich Hertel, Luis G. Quintanilla, Marcus Lehnert, Mattia Schmid, Davit Kharazishvili, Susan Fawcett, Michael Kessler

**Affiliations:** ^1^ Department of Systematic and Evolutionary Botany, University of Zurich, Zurich, Switzerland; ^2^ Albrecht von Haller Institute for Plant Sciences, University of Goettingen, Goettingen, Germany; ^3^ School of Environmental Sciences and Technology, University Rey Juan Carlos, Móstoles, Spain; ^4^ Geobotany and Botanical Garden Area, Herbarium, Martin-Luther-University Halle-Wittenberg, Halle, Germany; ^5^ Deputy Director of Research management of the Batumi Botanical Garden, Batumi, Georgia; ^6^ University and Jepson Herbaria, University of California, Berkeley, Berkeley, United States

**Keywords:** *Struthiopteris spicant*, ferns, arbuscular mycorrhizal fungi, environmental factors, mycorrhizal communities, AMF, facultative mycorrhizal plant, ITS metabarcoding

## Abstract

**Introduction:**

Ferns constitute the second largest group of vascular plants. Previous studies have shown that the diversity and composition of fern communities are influenced by resource availability and water stress, among other factors. However, little is known about the influence of these environmental factors on their biotic interactions, especially regarding the relationship between mycorrhizal fungi and ferns. The present study compares the mycorrhizal communities associated with 36 populations of Struthiopteris spicant L. Weiss across Europe and North America. This species exhibits a great tolerance to variations in light, nutrient, and pH conditions, and it can survive with and without mycorrhizae.

**Methods:**

With the aim of determining which environmental factors impact the composition and abundance of the root-associated fungal communities in this species, we used an ITS-focused metabarcoding approach to identify the mycorrhizal fungi present and analyzed the influence of climatic and edaphic variables at global and regional scales

**Results and discussion:**

We encountered striking differences in the relative abundance of arbuscular mycorrhizal fungi (AMF) between S. spicant populations at both spatial levels. We recorded a total of 902 fungal ASVs, but only 2– 4% of the total fungal diversity was observed in each individual, revealing that each fern had a unique fungal community. Light availability and the interactive action of pH and soil nitrogen concentration showed a positive influence on AMF relative abundance, explaining 89% of the variance. However, environmental factors could only explain 4– 8% of the variability in AMF community composition, indicating that it might be determined by stochastic processes. These results support the hypothesis that ferns may be more independent of mycorrhization than other plant groups and interact with fungi in a more opportunistic manner.

## Introduction

Ferns represent the second largest group of vascular plants with approximately 11.000 species distributed worldwide (PPG I, 2016). While some lineages diversified under nutrient-limited conditions around 400 my ago, others underwent more recent radiations after the rise of the angiosperms to ecological dominance, when most terrestrial ecosystems experienced nutrient enrichment (Berendse and Scheffer, 2009). Consequently, ferns can provide valuable information about the diverse strategies developed by plants to overcome the challenges of the colonization of land (Page, 2002). Although some of these strategies have been studied to some degree, others such as their biotic interactions have not received much attention yet (Page, 2002; Mehltreter et al., 2010; Mellado-Mansilla et al., 2022). In particular, the ability of ferns to form partnerships with mycorrhizal fungi is one of the least studied aspects of their ecology (Lehnert et al., 2017; Strullu-Derrien et al., 2018).

So far, two types of mycorrhiza have been observed in fern roots (Mehltreter et al., 2010; Lehnert et al., 2017): the less common fern-ericoid mycorrhizae formed by dark septate endophytes (DSE) and the common arbuscular mycorrhizae formed by arbuscular mycorrhizal fungi (AMF). DSE are usually classified as members of orders Heliotales and Pleosporales (phylum Ascomycota) but have also been found in Capnodiales, Eurotiales, Hypocreales, Sordariales, and Xylariales (Jumpponen, 2001; He et al., 2019; Malicka et al., 2022). They are characterized by the development of melanized hyphae and microsclerotia within the plant root cells (Jumpponen, 2001). Despite having a worldwide distribution and being very abundant in arid and contaminated environments (He et al., 2019; Huo et al., 2021; Malicka et al., 2022), little is known about the nature of plant-DSE associations (Malicka et al., 2022). Studies in angiosperms showcase DSE as mycorrhizal partners improving the adaptation of plants to drought, high salinity, toxic, and nutrient-poor conditions (Rodriguez et al., 2009; He et al., 2019; Malicka et al., 2022). However, their role in ferns remains poorly understood (Lehnert et al., 2017). Since DSE are often found growing within fern roots, especially in the absence of AMF (Rodriguez et al., 2009; Muthuraja et al., 2014; Lehnert et al., 2017), it has been suggested that this might reveal a potential shift in symbiotic allies during fern evolution (Lehnert et al., 2017). To our knowledge, there have been no studies exploring the relationships between DSE and ferns using molecular tools.

AMF form the oldest and most common type of mycorrhiza (Brundrett, 2004; Strullu-Derrien et al., 2018). These obligate symbionts included in the phylum Glomeromycota (Wijayawardene et al., 2022) develop hyphae that penetrate the root cortical cells and form arbuscules (involved in nutrient exchange) and, in certain lineages, vesicles (storage organs) (Brundrett, 2002, 2004; Strullu-Derrien et al., 2018). The mycorrhizal symbiosis is usually beneficial for both the fungus and the host, with the first obtaining plant-assimilated carbohydrates and lipids (Brundrett, 2004; Bennett & Klironomos, 2019), and the latter experiencing an increase in nutrient (phosphorus, nitrogen, potassium and copper) and water uptake, pathogen resistance, and endurance in toxic substrates (Brundrett, 2002, 2004; Jansa et al., 2008). AMF are prevalent in most terrestrial ecosystems establishing associations with around 87% of angiosperm and 100% of gymnosperm species (Brundrett, 2002, 2017). However, the percentage of AMF presence is lower in ferns, at approximately 67% (Lehnert et al., 2017). It has been suggested that the relatively low percentage of mycorrhization in ferns stems from limited photosynthetic capabilities, so that their growth may be more strongly limited by carbon rather than nutrients (Kessler et al., 2014; Guillen-Otero et al., 2024). However, the majority of AMF research has been conducted on angiosperms, leaving ancient plant lineages like ferns poorly studied (Pressel et al., 2016; Lehnert et al., 2017; Strullu-Derrien et al., 2018; Field et al., 2019). Thus, information regarding the composition and functionality of AMF communities associated with ferns remains scarce (Redecker et al., 2000; Wang and Qiu, 2006; Pressel et al., 2016; Lehnert et al., 2017; Strullu-Derrien et al., 2018).

Although numerous studies have optically screened fern roots to observe their mycorrhizae (e.g., Kessler et al., 2010, 2014; Muthuraja et al., 2014; Guzmán-Cornejo et al., 2023), only a few have used high-throughput sequencing to identify the root-associated fungal assemblages (Pressel et al., 2016; Lehnert et al., 2017; Sandoz et al., 2020; Guillen-Otero et al., 2023). In consequence, there is a lack of understanding of the fungal taxonomic diversity and their functionality in ferns (Lehnert et al., 2017; Strullu-Derrien et al., 2018; Sandoz et al., 2020; Guillen-Otero et al., 2023). Moreover, while it is well known that fern communities are impacted by precipitation, resource availability, and temperatures, among other factors (Kessler et al., 2011; Weigand et al., 2020), there is limited data related to the influence of environmental factors on fern-fungus interactions (Lehnert et al., 2017; Sandoz et al., 2020; Weigand et al., 2022; Guillen-Otero et al., 2024). In seed plants, the distribution, composition, and abundance of their associated AMF communities has been found to be influenced by temperature, precipitation, light availability, soil vertical structure, pH and nutrient concentration, biotic interactions, and the host and the fungus genotypes (Hodge et al., 2000; Öpik et al., 2006; Lekberg et al., 2007; Dumbrell et al., 2010b; Liu et al., 2014; Shi et al., 2014; Tedersoo et al., 2014; Bennett and Klironomos, 2019; Větrovský et al., 2019; Neuenkamp et al., 2021; Peña Venegas et al., 2021; Han et al., 2024; Lekberg et al., 2024). For example, low light conditions may restrict the plant photosynthesis and therefore its carbon production, causing a decline in AMF abundance and richness (Liu et al., 2014; Shi et al., 2014). On the other hand, phosphorus and nitrogen are essential for plant and fungi development and their concentration is affected by precipitation and soil pH, among other factors (Öpik et al., 2006; Hu et al., 2019), which highlights the complexity of this type of analysis.

Only one previous study has focused on the spatial variation of root-associated fungi in ferns (Sandoz et al., 2020). It documented AMF assemblages in the roots of *Botrychium lunaria* (Ophioglossaceae) using a metabarcoding approach, finding that soil pH, humus cover, and microbial connectivity influenced AMF diversity. However, environmental, and biotic factors only explained a small fraction of the variability, and the study covered a limited geographical area.

To better understand the geographical variation in the root-associated fungi in ferns, we aimed to compare the fungal communities associated with a specific fern species across its entire distribution range. We chose *Struthiopteris spicant* (L.) Weiss (Blechnaceae), which is distributed across Europe, northern Africa, the Macaronesian archipelagos, and North America (Molino et al., 2019). This terrestrial fern occurs in a very diverse range of habitats from acidic and sandy substrates to well-developed forest soils, and from sites fully exposed to the sun to localities in deep shade (Molino et al., 2021), providing the potential for diverse mycorrhizal associations. A recent greenhouse experiment exploring the response of *S. spicant* and its root-associated fungi revealed that these associations were facultative and largely determined by light availability (Guillen-Otero et al., 2024). These findings suggest that the growth of *S. spicant* is carbon-limited and mycorrhizae are only present when the plant have a carbon surplus. In the controlled environment, the plant’s sensitive response to changes in light availability led to shifts in the diversity and abundance of the associated AMF communities (Guillen-Otero et al., 2024).

In the present study, we aimed to characterize for the first time the root-associated fungal communities linked to natural populations of *Struthiopteris spicant* across Europe and North America using a metabarcoding approach (Guillen-Otero et al., 2023), to address the following questions: a) Do populations of *S. spicant* living under different environmental conditions share similar fungal assemblages? b) Which environmental factors determine the composition and abundance of these fungal communities?

## Materials and methods

### Collecting sites and sampling protocol

**Figure 1:**
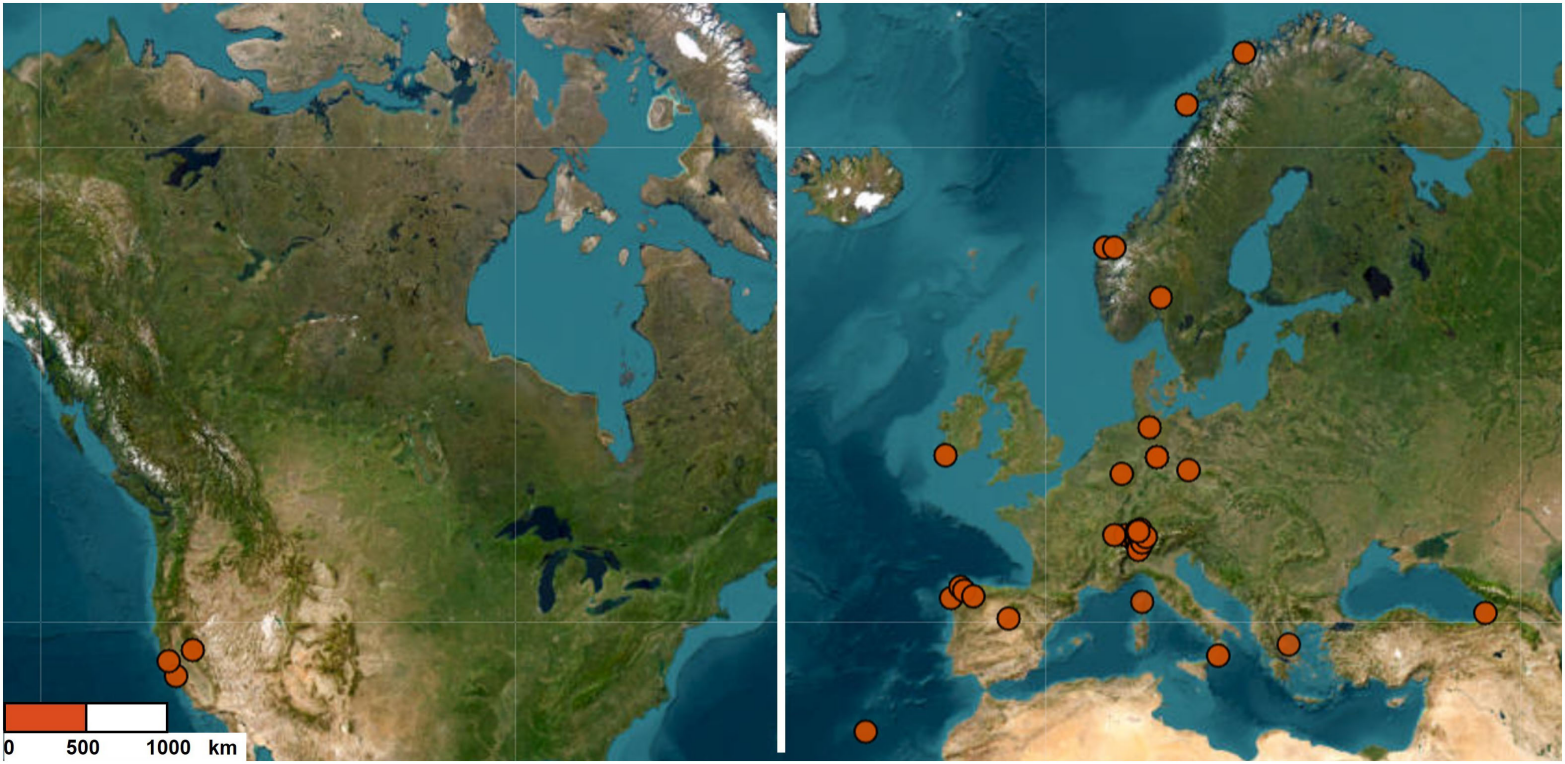
Populations sampled across the geographical range of *Struthiopteris spicant*.

We studied the fungal communities associated with 36 populations of *Struthiopteris spicant* located in 11 countries (**Figure 1**). The populations were geographically distant (50–8000 km) and thriving under very different environmental (humidity, soil type, light incidence, etc.), and plant (vitality, fertility) conditions (**Supplementary Figure S1**).

Our study was carried out at two spatial scales: across the entire distribution range of the species (here referred to as global level) and at higher density in Switzerland (regional level). The global analysis included 27 populations from Europe (including 3 Swiss ones) and the United States for a total of 79 individuals, with an elevation range between 15 m and 1800 m above sea level, 471 - 2976 mm of annual precipitation, and mean annual temperatures between -3°C and 19°C. The regional analysis focused on 12 populations, 9 of them not included in the global analysis, with a total of 35 individuals (elevation: 336 – 1691 m; precipitation: 688 – 2461 mm per year; temperatures: -3°C – 16°C). To explore the main drivers of differences among root-associated mycorrhizal fungal communities, we gathered climatic, edaphic, and vegetation data for each population.

The sampling included three plants per site with a minimal distance of 4 m from each other. From each individual we collected a mature leaf to analyze the leaf nutrient concentration and approximately 20 fragments of 4–6 cm to identify the dominant fungal partners. Samples were placed in labeled paper bags with silica gel for their rapid desiccation.

To measure the nutrient concentration in the soil (carbon (C), nitrogen (N) and phosphorus (P)), we collected 50–100 g of substrate directly between the roots of each specimen. The soil was placed in a labeled plastic bag and air-dried to avoid fungal proliferation. Leaf and soil samples were processed at the Albrecht von Haller Institute for Plant Sciences, University of Göttingen, Germany. For the soil, the pH_(H2O)_ value was measured using a 1:10 humus/water suspension after 24 h of equilibration, and the pH_(KCl)_ was measured subsequently after adding 1.86 g KCl to the suspension (corresponding to a 1N KCl solution).

For both leaf and soil material, the concentration of P was measured using the ICP-OES technique (inductively coupled plasma optical emission spectrometry, iCAP 7000, Thermo Fisher Scientific, Germany), after digestion of the material with 65% HNO_3_ at 195°C for 8 h. The total organic C and N concentrations were determined utilizing a C/N elemental analyzer (Vario EL III, Hanau, Germany).

We took a vertical photograph above each plant to estimate the vegetation density and hence the illumination received by the specimen. Photographs were analyzed using ImageJ software (Java 1.8.0_172, USA). In brief, we converted images into binary data, and established a local threshold using Phansalkar method to estimate the percentage of the image corresponding to black and white pixels respectively (Abramoff et al., 2004). The latter was used as a proxy of light incidence.

Finally, we extracted the values for bioclimatic variables bio1 (mean annual daily mean air temperatures averaged over 1 year) and bio12 (accumulated precipitation amount over 1 year) from the global CHELSA database (V2.1-V1.2, Karger et al., 2017).

#### Molecular analysis

We screened the root-associated fungi in the collected individuals using the protocol proposed by Guillen-Otero et al. (2023) to extract and purify fungal DNA in the roots of ferns and lycophytes. Briefly, samples were submerged for 2–3 minutes in liquid nitrogen and subjected to 3 cycles of 3 minutes/24 Hz in a TissueLyser II (QIAGEN, Hilden, Germany), using 5 mm stainless steel beads for mechanical disruption. We used the DNeasy Plant Mini Kit, following the manufacturer’s Quick-Start Protocol (QIAGEN, Hilden, Germany) and adapted by Guillen-Otero et al. (2023) to extract the fungal DNA from the dry roots. We purified the resulting samples with the Monarch Genomic DNA Purification Kit (New England Biolabs, Frankfurt am Main, Germany), quantified the resulting DNA with a NanoDrop One (Thermo Fisher Scientific, Basel, Switzerland), and obtained samples with the maximum purity (260/280 nm ∼1.8). We targeted the ITS rRNA region because it allows to retrieve an adequate representation of the general fungal community and also explore the arbuscular mycorrhizal fungi in particular (Guillen-Otero et al., 2023). We did not use a sequencing approach specifically targeting only AMF fungi because although this might provide better resolution within this fungal group, our aim was to obtain a broader perspective of the root-associated fungi. In particular, we used the percentage of AMF sequences relative to all fungal sequences obtained for each fern individual as a proxy for the intensity of mycorrhization, which would have been impossible using AMF-specific primers. This approach was previously successfully used in the greenhouse experiment of *S. spicant* by Guillen-Otero et al. (2024).

The ITS amplification was performed by EzBiome using the primer pair ITS1F (5’ CTTGGTCATTTAGAGGAAGT AA) (Gardes and Bruns, 1993)/ITS4: (5’ TCCTCCGCTTATGATATGC) (White et al., 1990) containing Illumina adapter overhang nucleotide sequences: In brief, each 25 μL of reaction included 12.5 ng of sample DNA, 12.5 μL 2x KAPA HiFi HotStart ReadyMix (Kapa Biosystems, Wilmington, MA) and 5 μL of 1 μM of each primer. The PCR protocol had an initial denaturation step performed at 95°C for 3min followed by 25 cycles of denaturation (95°C, 30 s), annealing (55°C, 30 s) and extension (72°C, 30 sec), and a final elongation of 5 min at 72°C. The PCR product was cleaned up with Mag-Bind RxnPure Plus magnetic beads (Omega Bio-tek, Norcross, GA), and a second index PCR amplification was performed using the same master mix conditions as described above. Cycling conditions included denaturation (95°C for 3 minutes, followed by 8 cycles of (95°C, 30 s), annealing (55°C, 30 s) and extension (72°C, 30 sec) and a 5 minutes’ elongation step at 72°C. The resulting product had 500–580 bp. DNA concentration was measured using the QuantiFluor dsDNA System on a Quantus Fluorometer (Promega, Madison, WI, USA). Libraries were normalized with the Mag-Bind^®^ EquiPure Library Normalization Kit (OmegaBio-tek, Norcross, GA). The pooled libraries were examined using an Agilent 2200 TapeStation and sequenced (2 x 300 bp paired end read setting) on the MiSeq (Illumina, San Diego, CA).

#### Bioinformatics

Data generated from the amplicon sequencing was processed following a quality control pipeline with dada2 package (Callahan et al., 2016; v1.22.0) in R (R Development Core Team; v4.1.2). Demultiplexed paired-end reads were checked for presence of primers and adaptors and filtered by sequence quality discarding those sequences with expected errors greater than 2 and length and keeping a minimal length of 450 bases. We estimated the error rates by sample. By means of a minimum overlap of 4 bases and a maximum mismatch of 2 bases sequences were dereplicated, denoised and merged. The resulting data was used to build an amplicon sequence variant table (ASV) in which chimeric sequences were identified and removed. We completed the taxonomic assignment applying the naïve Bayesian classifier (RDP classifier) with the curated fungal reference sequences from UNITE (Abarenkov et al., 2024; v8.3) and following the classification of the Kingdom Fungi proposed by Wijayawardene et al. (2022). After filtering out unidentified sequences at phylum level, we obtained the core fungal mycobiome by specimen (hereby core mycobiome), by removing the taxa with a relative abundance lower than 1% in each sample as described by Guillen-Otero et al. (2023). The metadata and the taxonomic classification and ASVs abundance for the general core microbiome dataset can be found in S1.

#### Statistical analysis

All the statistical analyses were executed in R (R Development Core Team; v4.1.2). The representativeness of our sample size was tested through a rarefaction analysis (rarecurve function, vegan package, v 2.6.4) and the relative composition of the fungal communities at phylum level and AMF (arbuscular mycorrhizal fungi) communities at family level was summarized with ggplot package (v 3.4.2). The following analyses were carried out at global and regional level.

We conducted an additive diversity partitioning analysis (β = γ – α) to quantify the contribution of individuals and populations to the overall fungal diversity. Calculations were carried out in Microsoft Excel where gamma-diversity (γ) is the cumulative diversity within each fern population (γ = fungal species total), alpha-diversity (α) is the average diversity per specimen (α1 =fungal species average per fern, α2 =fungal species average per population), and beta-diversity (β) is the diversity among samples by population (β1 = α2 – α1, and β2 = γ – α2) (Veech et al., 2002).

We tested the effects of environmental conditions (light incidence, mean annual temperature, annual precipitation, soil pH, and the concentration of carbon, nitrogen and phosphorus in the soil) on the AMF species richness, represented as the sum of ASVs in a sample, and its abundance, represented as the total number of sequences in a sample, using a one-way Analysis of Variance (ANOVA) with Bray–Curtis dissimilarity as measure (adonis, vegan package, v 2.6.4). The Shapiro-Wilk test and Bartlett’s test were previously used to determine the normality and homogeneity of variances in the data, and both requirements were met.

We analyzed the relationship between environmental factors (light, mean annual temperature, annual precipitation, soil phosphorus concentration, soil nitrogen concentration and soil pH) and the richness and relative abundance of AMF by population using a regression simple analysis (lm function, stats package). However, linear relationships are highly improbable among such complex variables therefore we built a Generalized Additive Model (GAM) to evaluate their suitability to predict AMF abundance and richness in *S. spicant* (mgcv package, v1.9.0).

Finally, to visualize the influence of environmental factors in AMF communities associated with *S. spicant* we performed a Canonical Correspondence Analysis (CCA), using the function cca (vegan package, v 2.6.4).

## Results

The analysis of the mycorrhizal communities associated with the 105 fern individuals yielded 503.323 reads corresponding to 902 ASVs (amplicon sequence variants) of the core mycobiomes (taxa representing more than 1% of the total abundance by sample). Arbuscular mycorrhizal fungi (AMF, phylum Glomeromycota) accounted for 464 ASVs (12.278 reads) out of this total.

**Figure 2:**
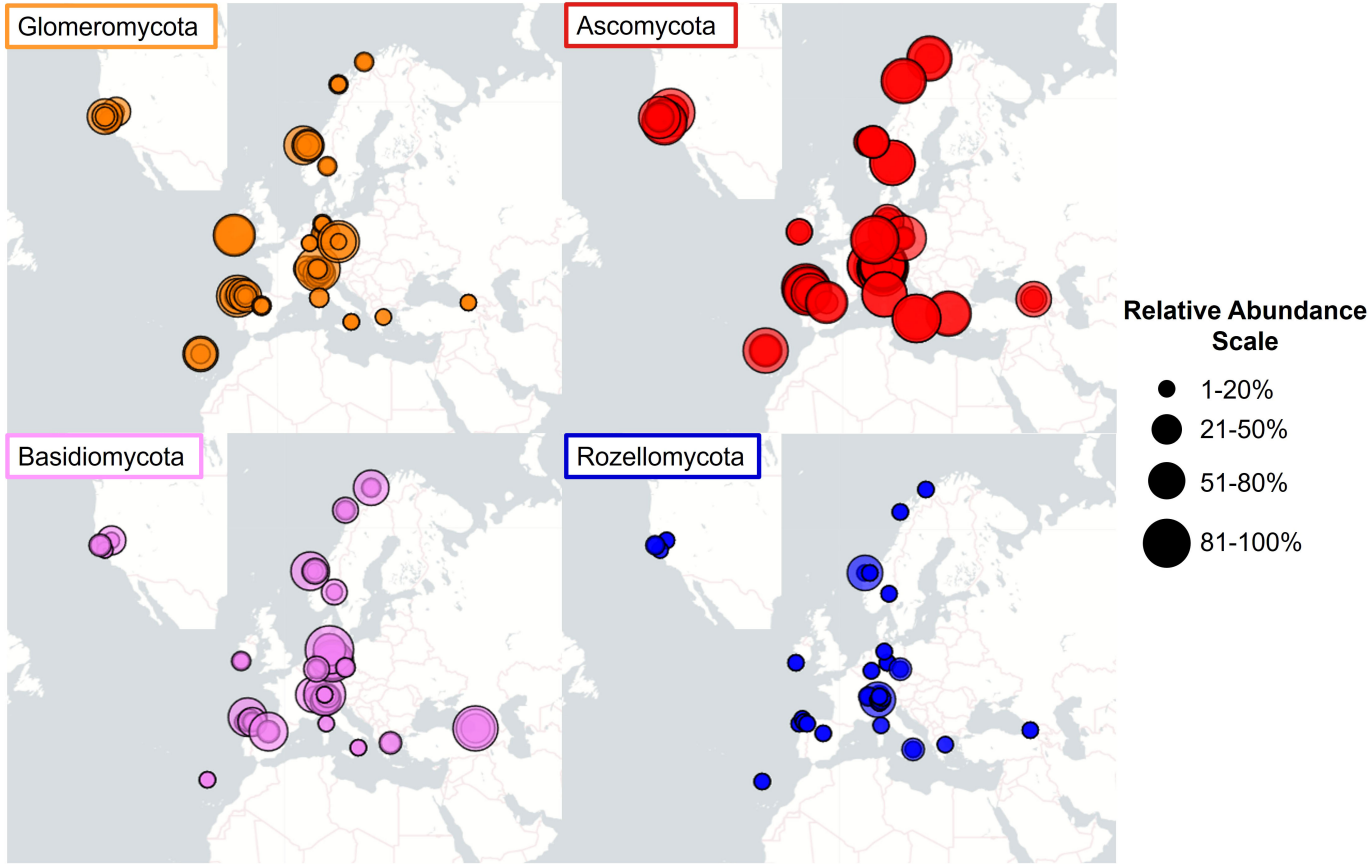
Maps of relative abundances of the four main phyla in fungal communities associated to *Strupthiopteris spicant* in 36 natural populations across Europe and North America.

We found a great variability in the relative abundance of the fungi linked to *Struthiopteris spicant* populations. Fungal communities were dominated by the phyla Ascomycota, Glomeromycota, Basidiomycota, and Rozellomycota, with clearly differentiated geographic abundance patterns (**Figure 2**). In this analysis we included all the studied populations because we encountered similar patterns at global and regional levels. The relative abundance of Glomeromycota, measured as the percentage of ASVs assigned to this phylum, varied from 1% to 80% and was significatively different among populations (F=2.64, df = 35, P<0.0001), being highest in the central part of its European range and decreasing toward the northern and southern distribution limits (**Figure 2**). In contrast, Ascomycota were well-represented across all sites (25–90%). Basidiomycota and Rozellomycota showed a more irregular pattern, reaching relative abundances between 10–80% and 1–60%, respectively. As reported in previous studies (Guillen-Otero et al., 2023, 2024), among the Glomeromycota there was a predominance of members of the family Glomeraceae, specifically the genus *Glomus*, with *Glomus macrocarpum* being the species shared by all the samples containing AMF. Regarding the non-mycorrhizal taxa, the orders Pleosporales, Heliotales (Ascomycota), and Tremellales (Basidiomycota) were the most represented across the samples.

**Figure 3:**
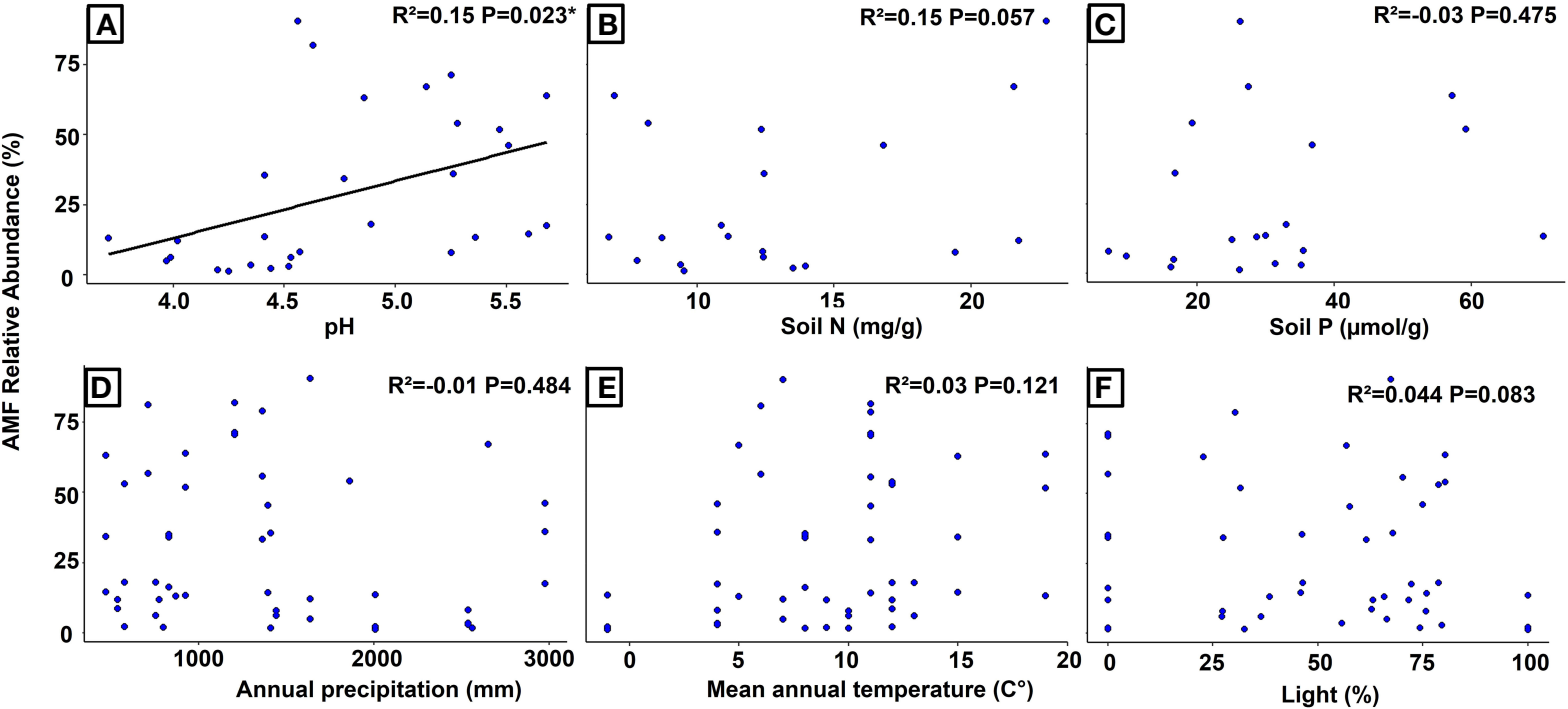
Regression analyses showing the relationships between the relative abundance of AMF (arbuscular mycorrhizal fungi) and different environmental variables in *Strupthiopteris spicant* across its distribution range. **(A)**: pH, **(B)**: Soil Nitrogen (mg/g), **(C)**: Soil Phosphorus (µmol/g), **(D)**: Annual Precipitation (mm), **(E)**: Mean Annual Temperature (°C), **(F)**: Light (%).

**Figure 4:**
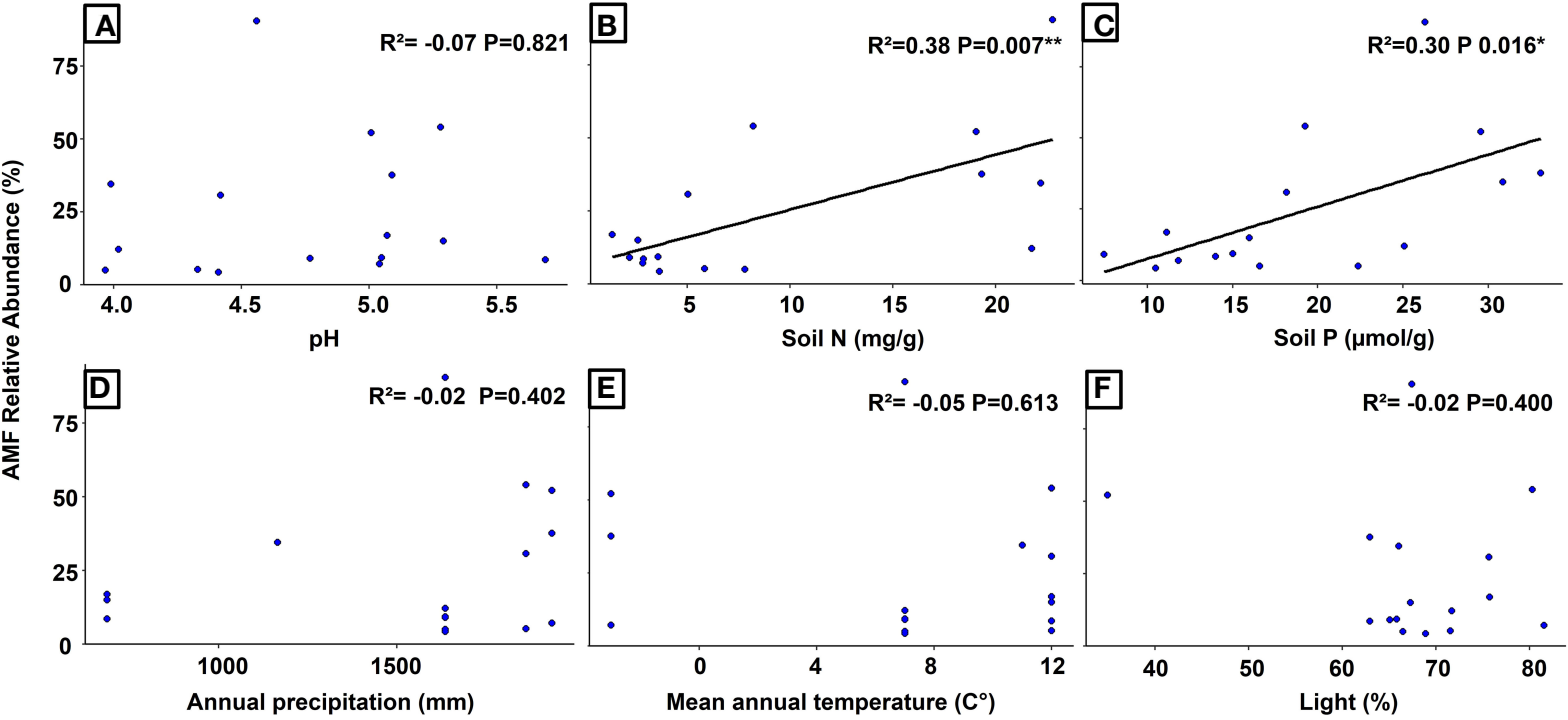
Regression analyses showing the relationships between the relative abundance of AMF (arbuscular mycorrhizal fungi) and different environmental variables in *Strupthiopteris spicant* populations in Switzerland. **(A)**: pH, **(B)**: Soil Nitrogen (mg/g), **(C)**: Soil Phosphorus (µmol/g), **(D)**: Annual Precipitation (mm), **(E)**: Mean Annual Temperature (°C), **(F)**: Light (%).

The simple regression analysis revealed a significant positive relationship between the relative abundance of AMF and soil pH at the global level (**Figure 3**), whereas the analysis of Swiss populations found significant positive relationships with the concentrations of nitrogen and phosphorus in the soil (**Figure 4**). Regarding AMF richness, we found mean annual temperature, light availability (**Supplementary Figure S2**), and once more the soil pH (**Supplementary Figure S3**) to be the most significant factors. Coincidentally, the Generalized Additive Model (GAM) found light availability (R²adj. = 0.758 and R²adj. = 0.876, *P* < 0.001) and the interactive action of pH and soil nitrogen concentration (R²adj. = 0.758, *P* < 0.001) as the key predictors of AMF abundance, explaining 89.4% of the variance (**Table 1**), and richness with 90.4% of explained variance (**Table 2**). When including only populations at the regional level, the GAM was inconclusive.

**Table 1:** Generalized Additive Model representing the relationship between five environmental factors and the relative abundance of arbuscular mycorrhizal fungi in *Struthiopteris spicant* populations.

Variables	Chi.sq	p-value	UBRE	R-sq.(adj)	Deviance explained
Mean annual temperature	3.065	0.832	3.7422	0.758	89.4%
Annual precipitation	0.042	0.839			
Soil nitrogen	0	0.987			
pH	0.001	0.973			
Light	103.106	< 2e-16 ***			
pH * Soil Nitrogen	72.939	< 2e-16 ***			
Mean annual temperature* Annual precipitation	6.156	0.901			

Asterisks indicate the levels of statistical significance: * p<0.05, *** p<0.001.

**Table 2:** Generalized Additive Model representing the relationship between five environmental factors and the richness of arbuscular mycorrhizal fungi in *Struthiopteris spicant* populations.

Variables	Chi.sq	p-value	UBRE	R-sq.(adj)	Deviance explained
Mean annual temperature	0.325	0.568441	1.0232	0.876	90.4%
Annual precipitation	0.113	0.736737			
Soil nitrogen	0	0.993225			
pH	0.423	0.999219			
Light	31.884	< 2e-16 ***			
pH * Soil nitrogen	22.104	0.225593			
Mean annual temperature* Annual precipitation	17.530	0.243927			

Asterisks indicate the levels of statistical significance: * p<0.05, *** p<0.001.

The additive partitioning analysis allowed us to quantify the contribution of each component to the arbuscular mycorrhizal fungi diversity at the global and regional scales. At the global level, the average AMF diversity of each fern individual represented approximately 1.9% (α_1 =_ 12) of the total number of ASVs recorded (γ=659). As expected, we encountered a certain overlap in fungal presence between fern specimens, particularly within populations (α_2 =_ 9.3%). The ASV shared by the highest number of fern individuals was recovered in 31 out of 79 plants (39%). The fungal community composition was variable between populations (β_2_) contributing the most to AMF diversity, at approximately 95%. In the Swiss populations, we found 4.2% of the total number of ASVs recorded (γ=310) per individual (α_1 =_ 13). The ASV shared by the highest number of fern individuals was recovered in 13 out of 26 plants (50%), whereaS28 ASVs were only found in a single fern specimen.

**Figure 5:**
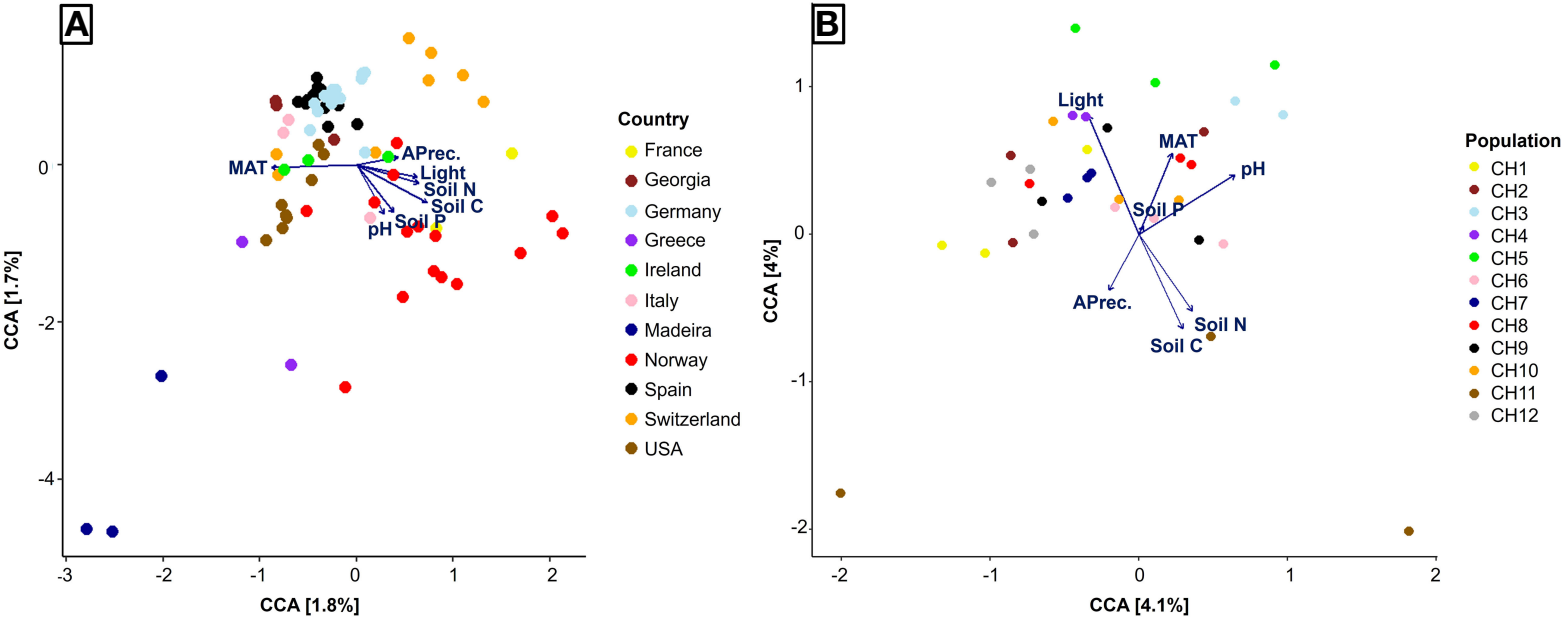
Canonical Correspondence Analysis of fungal communities associated to *Strupthiopteris spicant* in 27 natural populations across Europe and North America **(A)**, and 12 populations in Switzerland **(B)**. MAT, Mean annual temperature; APrec, Annual precipitation; Light, Light availability; Soil N, Soil nitrogen concentration; Soil P, Soil phosphorus concentration; Soil C, Soil carbon concentration; pH, Soil pH.

Finally, on the global scale, the Canonical Correspondence Analysis (CCA) explained less than 4% of the variance in fungal community composition. We observed a strong geographical pattern that corresponded to a gradient of MAT in one direction, opposed by all the remaining factors (**Figure 5A**). When we reduced the geographical effect by including only Swiss populations in the CCA, the explained variance increased to about 8%. In this case, the most influential factors were light availability, soil pH, and soil N and C concentrations (**Figure 5B**).

## Discussion

Most studies examining fern-fungi relationships in natural ecosystems have relied on visual inspection of the roots to confirm the presence of mycorrhizal partners (Lehnert et al., 2017; Strullu-Derrien et al., 2018). The recent development of molecular methods has stimulated the interest in understanding the nature and ecology of these alliances (Öpik et al., 2006; Pressel et al., 2016; Lehnert et al., 2017; Strullu-Derrien et al., 2018; Field et al., 2019). Still, the identity of the fungal assemblages associated with fern roots remains largely unknown (Strullu-Derrien et al., 2018). Only a few studies have analyzed the influence of environmental factors on fern-fungi relationships either through greenhouse experiments (Guillen-Otero et al., 2024), field experiment (Weigand et al., 2022; Guillen-Otero et al., 2024), the comparison of different species and/or habitats (Kessler et al., 2014; Perez-Lamarque et al., 2022), or by analyzing a particular taxon in an area of interest, e.g., the Alps (Sandoz et al., 2020). In the present study, we used a metabarcoding approach to analyze 105 root samples from 36 populations of *Struthiopteris spicant*, obtaining 902 ASVs (amplicon sequence variants) from which 462 ASVs were assigned to Glomeromycota (AMF). Summarizing, our results indicate that the composition of arbuscular mycorrhizal fungi (AMF) communities varies greatly among *S. spicant* populations, and that although environmental factors such as light availability, soil pH, and soil nitrogen (N) concentration can partially explain the abundance and richness of AMF, they cannot be used to predict the taxonomic composition of these mycorrhizal communities.

The challenge of relating the variation observed in fungal assemblages to environmental and biotic variables is well known from previous studies on angiosperms (Lekberg et al., 2007; Dumbrell et al., 2010b; Talbot et al., 2014; Caruso, 2018). In some cases, this difficulty might be related to the study design, such as an inefficient sampling structure, a low number of replicates, or the need to incorporate additional environmental variables into the analysis (Caruso, 2018). In our study, we collected fine roots from three plants per population with a minimal distance of four meters from each other to avoid fungal community sharing. We sampled a total of 36 sites covering most of the distributional range of *S. spicant*, spanning different elevations, soil types, environmental conditions, and plant status of vitality, fertility, and population size. Moreover, the rarefaction curves which represented ASV number as a function of the sequenced reads per sample, reached a saturation point after a low number of reads (**Supplementary Figure S6**). This suggests that the sequencing depth was adequate to analyze the core fungal microbiome (mycobiome) associated with *S. spicant*, as confirmed by prior studies in ferns using the same metabarcoding approach (Guillen-Otero et al., 2023, 2024). It is important to consider that the sequencing method used during this study can have a selective bias toward certain taxa (Manter and Vivanco, 2007). However, it is a standard method to study root-associated fungi (Rimington et al., 2018; Sandoz et al., 2020; Guillen-Otero et al., 2023, 2024). Our results are comparable and coincidental with prior reports and hence it is likely that sequencing biases do not play a strong role in their determination. With these details in mind, we consider our study design robust to representatively sample the diversity of root-linked fungi in *S. spicant*.

Climatic and edaphic factors have been reported as important drivers of mycorrhizal abundance and richness in angiosperms (Öpik et al., 2006; Tedersoo et al., 2014; Hu et al., 2019; Větrovský et al., 2019; Han et al., 2024). Still, fern-fungus relationships remain poorly explored (Gemma et al., 1992; Zhang et al., 2004; Kessler et al., 2014; Pressel et al., 2016; Lehnert et al., 2017; Sandoz et al., 2020; Perez-Lamarque et al., 2022; Guillen-Otero et al., 2023). Although other authors included variables such as habitat, soil vertical structure, and seasonality in their analyses (e.g., Dumbrell et al., 2010b; Öpik et al., 2006; Hu et al., 2019), previous studies comprising as many as 34 environmental variables have repeatedly indicated that temperature, precipitation, light, pH, and carbon (C), phosphorus (P), and nitrogen (N) availability in the soil appear to have the strongest influence on AMF community assemblages (Öpik et al., 2006; Dumbrell et al., 2010b; Tedersoo et al., 2014; Brundrett, 2017; Hu et al., 2019; Větrovský et al., 2019; Neuenkamp et al., 2021). We decided to include in the present analysis those factors that were most frequently encountered as significant for the fungal root microbiome or root mycobiome.

We observed a reduction in AMF abundance toward the northern and southern limits of the distribution of *S. spicant* in Europe. This might be related to the ecological preferences of AMF fungi which have a more restricted climatic tolerance than members of the Ascomycota and the Basidiomycota and a tendency to decline at higher latitudes (Hu et al., 2019). In addition, it is possible that the edaphic characteristics of a given locality favored the proliferation of non-AMF taxa coincidentally toward the upper and bottom limits of the range (Dumbrell et al., 2010a, 2010b; Tedersoo et al., 2014). Alternatively, it is possible that in these marginal population of *S. spicant* the fern individuals are at the limits of their ecological niche, so that they are physiologically stressed and unable to produce enough carbohydrates to engage in mycorrhizal interactions. This would be in accordance with a previous greenhouse experiment, where plants of *S. spicant* grown with low light and hence low photosynthetic gain, did not engage in mycorrhizal symbioses (Guillen-Otero et al., 2023).

We found light availability and the interaction between soil pH and soil N to be the main predictors of the relative abundance (**Table 1**) and richness of AMF (**Table 2**). In the greenhouse experiment we also found that light availability is the most important factor determining the relationships between *S. spicant* and its mycorrhizal partners (Guillen-Otero et al., 2024). Experiments in angiosperms have also previously reported a diminution of AMF biomass, colonization rate, and diversity when decreasing light availability (Liu et al., 2014; Shi et al., 2014). Together, this positive relationship between light availability and AMF relative abundance and richness suggest that a drop in the production of photosynthetically fixed carbon reduces the plant investment in an association that has turned negative from the plant perspective (Johnson et al., 2010; Wang et al., 2017; Lekberg et al., 2024). When the loss in C to the fungus exceeds the benefits obtained by the plant regarding phosphorus and nitrogen acquisition, it is compensated by an increment on the allocation of biomass to aboveground structures (Johnson et al., 2010; Neuenkamp et al., 2021). The abundance and richness of AMF associated to *S. spicant* thus appears to be mainly regulated by carbon limitation. Facultative mycorrhizal (FM) plants have the ability to regulate the association with AMF based on their needs and the current environmental conditions (Brundrett, 2009; Neuenkamp et al., 2021). This plasticity allows them to optimize their energetic investments depending on the functionality of the symbiosis, e.g., suppressing and preventing colonization when light is limited (Zobel et al., 2024). Ferns may have developed this strategy to expand into new areas without suitable fungal partners (Brundrett and Tedersoo, 2020; Moyano et al., 2020; Guillen-Otero et al., 2024; Zobel et al., 2024).

Soil pH has been recognized as a major structuring factor for AMF communities because it affects the extension of extraradical mycelia (Van Aarle et al., 2002), the N_2_ fixation (Schubert et al., 1990), the rates of decomposition (impacting the soil C/N ratio), and the solubility of phosphate compounds, therefore influencing AMF abundance and richness (Dumbrell et al., 2010b). As *S. spicant* inhabits soils with pH values between 3.7 and 5.7, AMF development might be limited by N availability, generating plant-fungal competition for this resource (Hodge et al., 2000; Hodge and Fitter, 2010).Thus, it is not unexpected to find that the concentrations of N and P in the soil were significant at the regional scale (Swiss populations), explaining 68% of the variability in AMF relative abundance and richness. However, contrasting with previous studies in angiosperms (Egerton-Warburton & Allen, 2000; Johnson et al., 2010; Dumbrell et al., 2010b; Liu et al., 2014; Johnson et al., 2015; Lekberg et al., 2024), we encountered a positive linear relationship between the concentration of these nutrient in the soil and relative abundance of AMF. Mycorrhizal fungi require relatively large quantities of N to grow (Johnson et al., 2015), raising the possibility that AMF nutrient requirements also play an important role in the plant response to colonization (Hodge et al., 2000; Neuenkamp et al., 2021; Han et al., 2024).

As indicated by the additive partitioning analysis, the largest portion of the diversity was accumulated at the population level, where we found striking differences in the composition of the AMF assemblages. On average, only 2% (at the global level) and 4% (at the regional level) of the total fungal diversity was found in each fern individual, showing that in essence each fern individual had a unique fungal community. This coincides with the almost nonexistent explanatory power of the ordination analyses (4–8% explained variance). Across geographically distant populations, we found that mean annual temperature had a slight effect on AMF community composition. Temperature and precipitation not only affect plant physiology and ecology (e.g., the uptake of C, P, and N) (Větrovský et al., 2019; Yuan et al., 2024) but also the interaction between fungal functional groups (Tedersoo et al., 2014).

When we considered just the Swiss populations, we found that the composition of AMF communities linked to *S. spicant* was mainly influenced by light, soil C and N, and soil pH. Together, this reveals a very low specificity of fungal partners in fern species, coinciding with previous findings (Perez-Lamarque et al., 2022; Guillen et al., 2024; Guillen-Otero et al., 2024). For instance, a regional study of *Botrychium lunaria* (Ophioglossaceae) in the Swiss Alps, found that the composition of the AMF community associated was significantly impacted by soil pH and humus cover but was not predicted by them (Sandoz et al., 2020). A study analyzing the response of AMF associated with two epiphytic and two terrestrial fern species to 15 years of N and P fertilization in Ecuador also showed that fertilization did not determine the composition of the fungal assemblages (Guillen et al., 2024). Random variation in the community composition might be introduced by taxon-specific constraints on dispersal (e.g., variations in the size and shape of the spores) and colonization processes at a regional scale (Bahram et al., 2015; Lekberg et al., 2024).

Among the AMF, members of the family Glomeraceae dominated most of the mycorrhizal communities, corresponding with the results of previous analyses focused on ferns (Sandoz et al., 2020; Perez-Lamarque et al., 2022; Guillen-Otero et al., 2023, 2024). Glomeraceae is the most widespread mycorrhizal family in terrestrial ecosystems (Oehl et al., 2010), capable of outcompeting other taxa in terms of fast growth, biotic stress control, and productivity enhancement (Powell et al., 2009; Maherali & Klironomos, 2012; Han et al., 2024). However, some *S. spicant* populations thriving under temperatures of 9–10°C and exhibiting low AMF abundances were dominated by taxa from the families Gigasporaceae and Acaulosporaceae. Gigasporaceae seems favored in areas where P availability is low because its large extraradical hyphae facilitates nutrient uptake to plants (Delroy et al., 2024; Han et al., 2024), whereas Acaulosporaceae has been documented as a slow root colonizer that easily coexists with other lineages (Maherali and Klironomos, 2012). Additional factors such as taxon-specific dispersal limitations (Tedersoo et al., 2014), and soil fertility (Öpik et al., 2006; Dumbrell et al., 2010b) also have an impact in the predominance of certain AMF taxa (Lekberg et al., 2007). For example, a field study addressing the role of niche restrictions and dispersal in the composition of AMF communities of maize (*Zea mays*) found that members of Glomeraceae are at a disadvantage when plants grow in sandy soil, whereas the abundance of Gigasporaceae is negatively correlated to the percentage of clay in the substrate (Lekberg et al., 2007). Furthermore, previous reports have shown that phylogenetically old plant lineages are mostly associated to AMF from the genus *Glomus* (Winther & Friedman, 2008; Field et al., 2015; Sandoz et al., 2020; Guillen-Otero et al., 2023). *Glomus macrocarpum* appeared to have distinct advantages over other fungal species as it was shared among all *S. spicant* populations that contained AMF. During greenhouse and field experiments in angiosperms, this taxon has shown significant effects in nutrient transfer, plant development, and pathogen resistance (Giri et al., 2005; Ghosh and Panja, 2021).

Non-AMF taxa from the Pleosporales, Heliotales (Ascomycota), and Tremellales (Basidiomycota) orders were especially predominant in populations with low presence of Glomeromycota (**Supplementary Figure S4**). Although these orders contain taxa that have been recognized as endophytes (Rungjindamai and Jones, 2024; Zeng et al., 2024), their functionality regarding ferns remains unknown. Dark septate endophytes (DSE) in particular are frequent intracellular root associates with plants and they have been commonly observed in fern roots (Kessler et al., 2010; Lehnert et al., 2017). Most of them have been classified as members of Heliotales and Pleosporales (Jumpponen, 2001; He et al., 2019; Malicka et al., 2022). It has been speculated that DSE may act as mycorrhizal partners in situations where AMF are absent (Kessler et al., 2010; Lehnert et al., 2017; He et al., 2019), even though some authors have found a positive correlation between the abundances of AMF and DSE, suggesting a diversification in functions (Malicka et al., 2022; Zeng et al., 2024).

To conclude, the present study explores for the first time the fungal communities associated with a single fern species across its entire distribution range. Developing the analysis at two different scales (global and regional) revealed the role of geographical factors when assessing the impact of environmental variables in root-associated fungi. Our results emphasize the complexity of the relationships between AMF and *Struthiopteris spicant*. While the relative abundance and richness of these fungal assemblages can be predicted based on light availability, pH level, and soil nutrient concentration, their composition appears to be differentially influenced by the host plant, the fungal community dynamic, and stochastic processes, which make it hard to predict using climatic, edaphic, and biotic measures. Moreover, the ecological success of *S. spicant* across such a variable range of conditions in Europe and North America emphasizes the advantages of the facultative mycorrhizal status in ferns (Moyano et al., 2020; Guillen-Otero et al., 2024).

## Data availability statement

The datasets presented in this study can be found in online repositories. The names of the repository/repositories and accession number(s) can be found in the article/**Supplementary Material**.

## Author contributions

TG: Data curation, Formal analysis, Investigation, Methodology, Project administration, Resources, Software, Validation, Visualization, Writing – original draft, Writing – review & editing. MK: Conceptualization, Formal analysis, Funding acquisition, Investigation, Methodology, Project administration, Resources, Supervision, Validation, Writing – review & editing. DH: Formal analysis, Writing – review & editing. LQ: Resources, Writing – review & editing. ML: Resources, Writing – review & editing. MS: Resources, Writing – review & editing. DK: Resources, Writing – review & editing. SF: Resources, Writing – review & editing.
