# Supplementary figures and images for: Comparative analysis of mycorrhizal communities associated with Struthiopteris spicant (L.) Weiss across Europe and North America
Source: Front Plant Sci. 2024 Jun 4;15:1402946. doi: 10.3389/fpls.2024.1402946 (PMC11186384; doi:10.3389/fpls.2024.1402946)

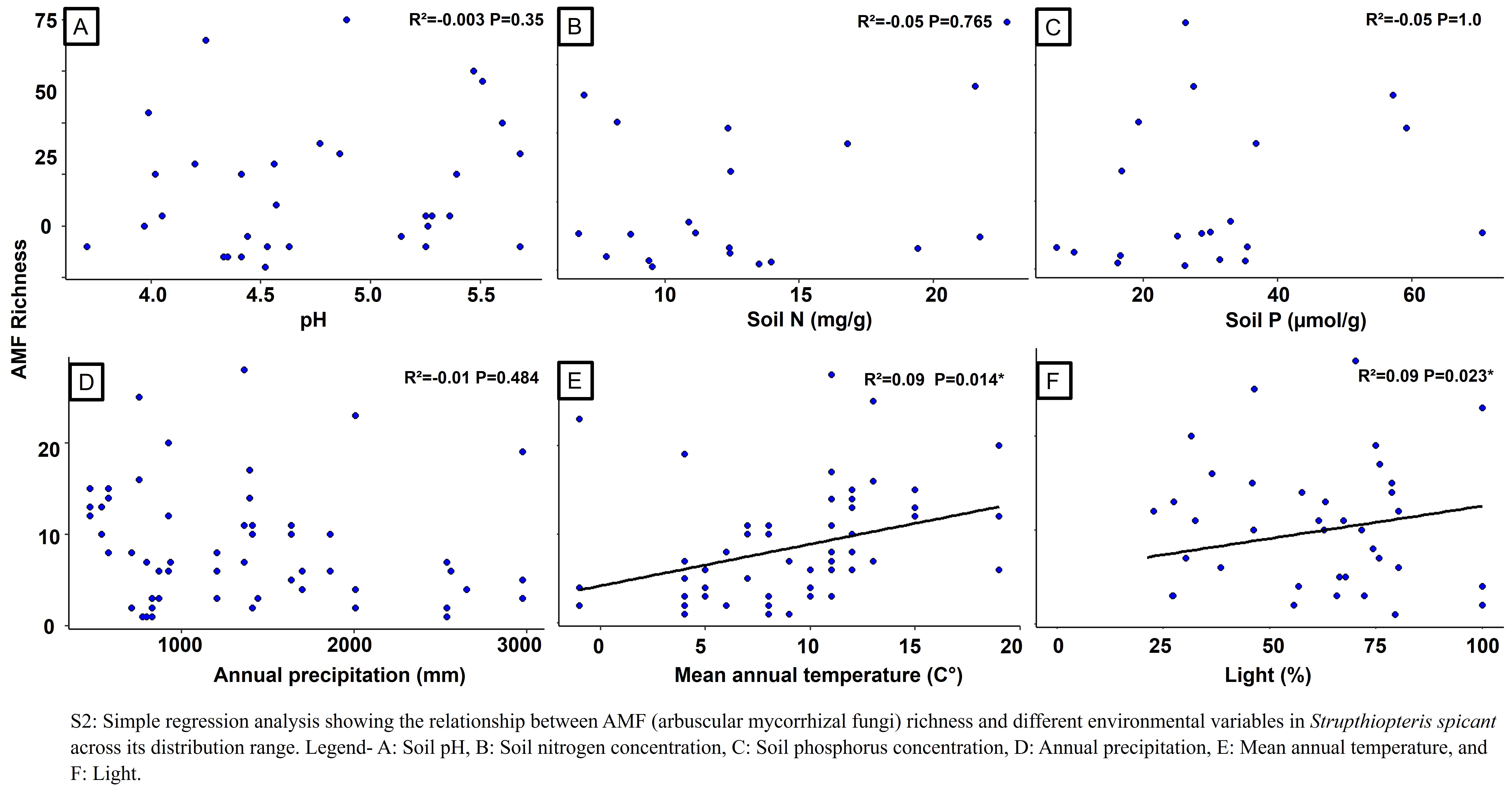

Supplement: Supplementary file 1 [file Image_1.jpeg]

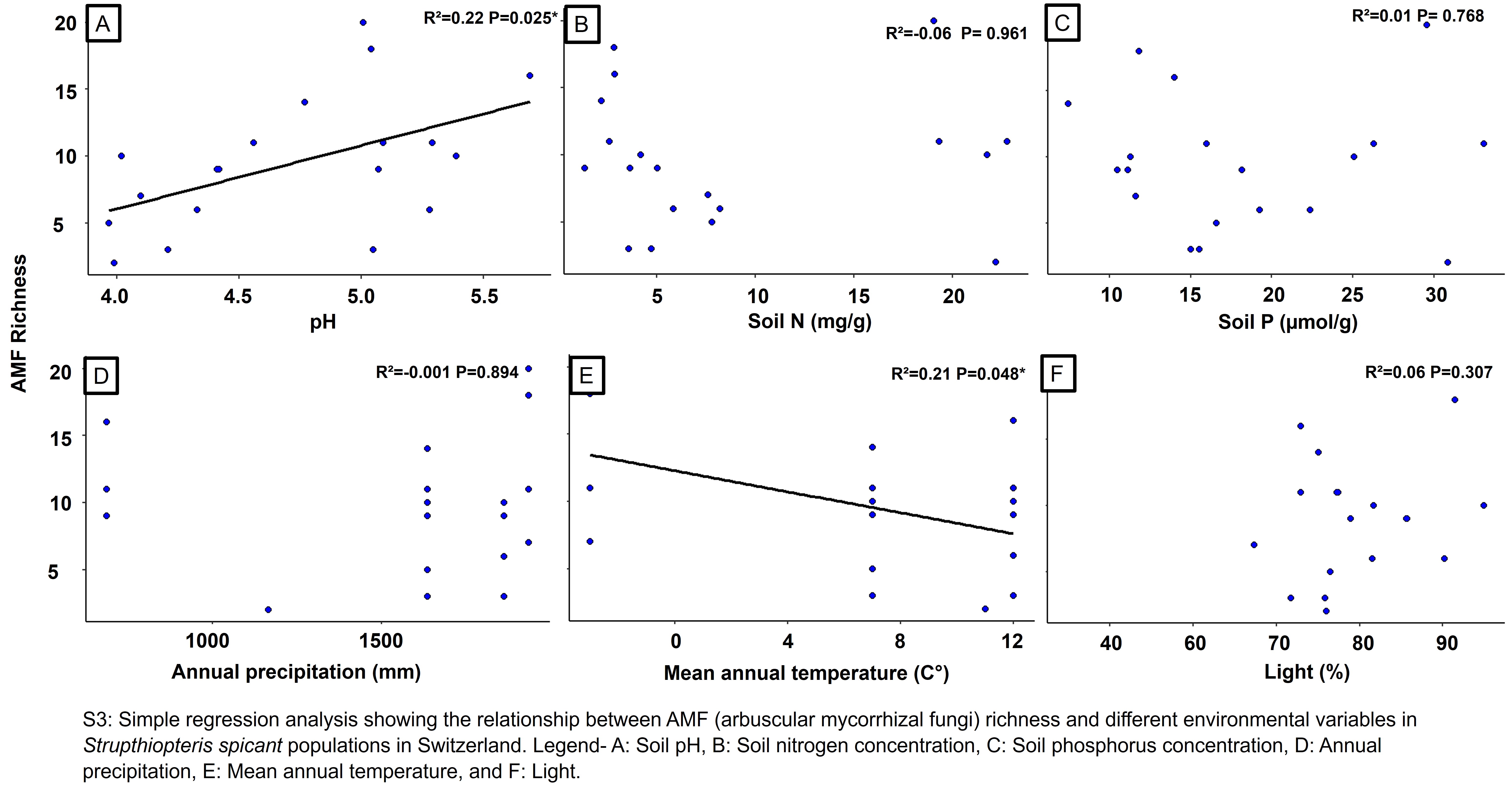

Supplement: Supplementary file 2 [file Image_2.jpeg]

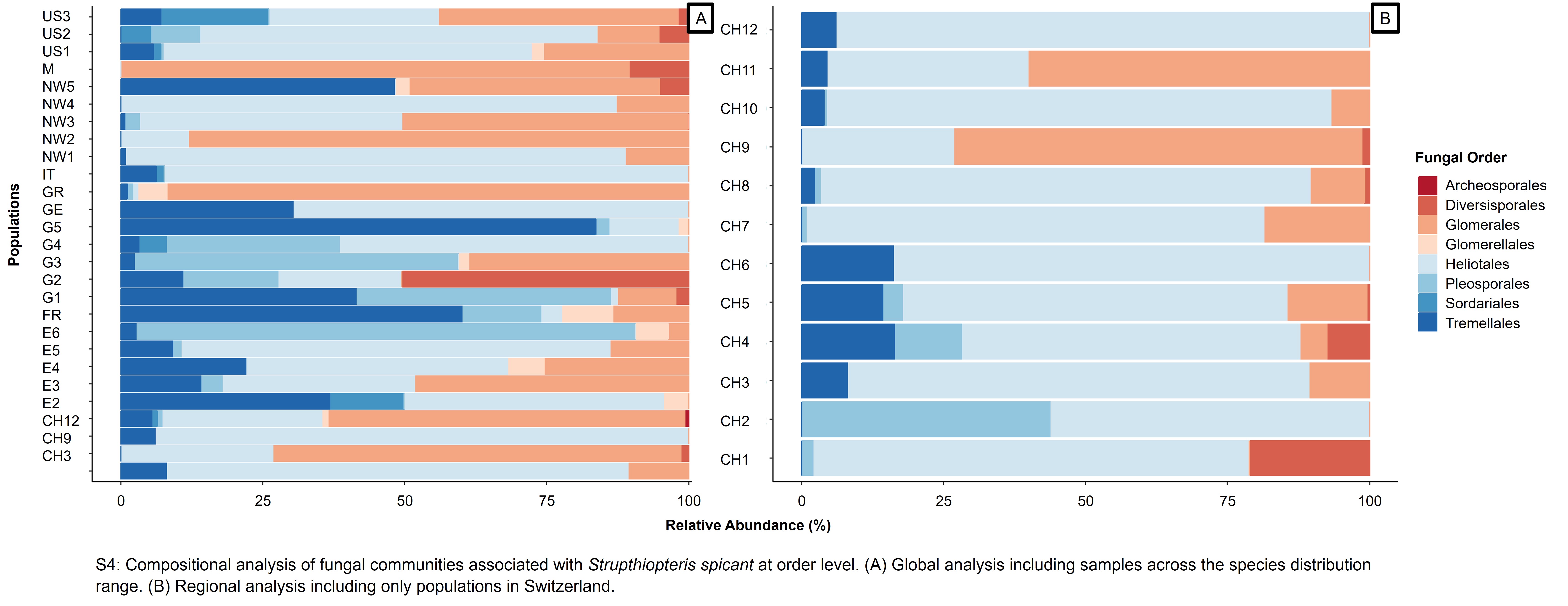

Supplement: Supplementary file 3 [file Image_3.jpeg]

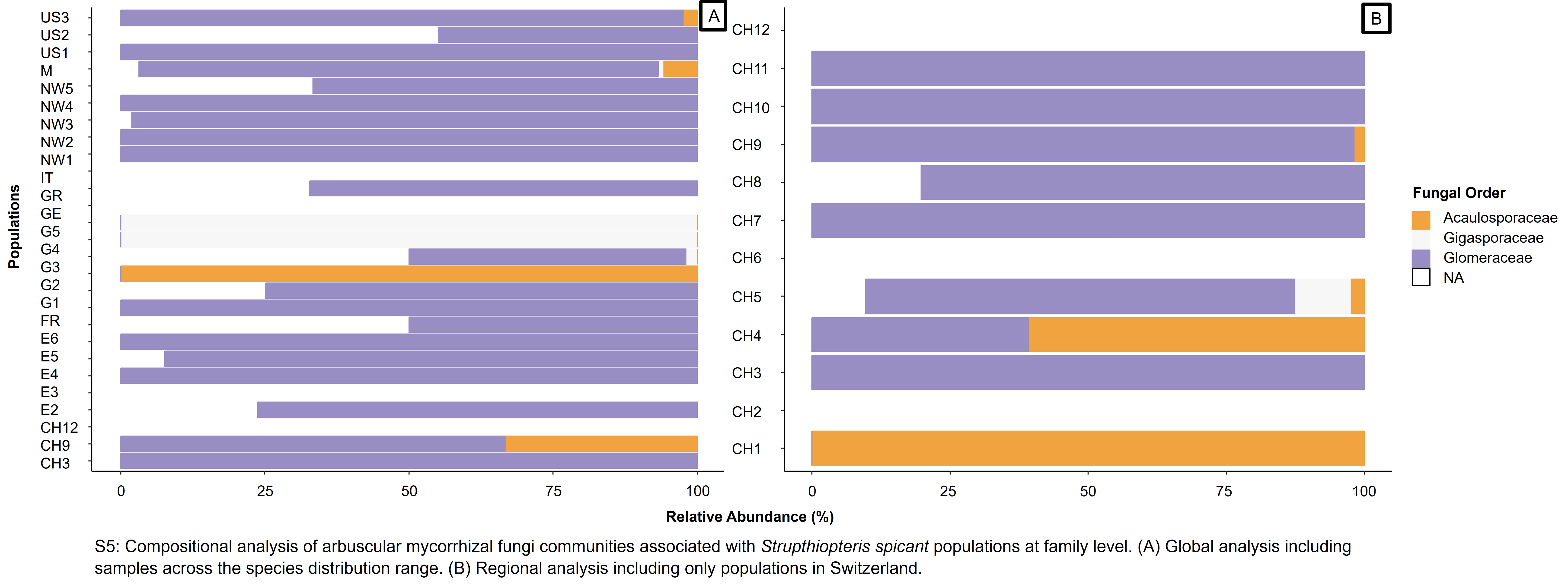

Supplement: Supplementary file 4 [file Image_4.jpeg]

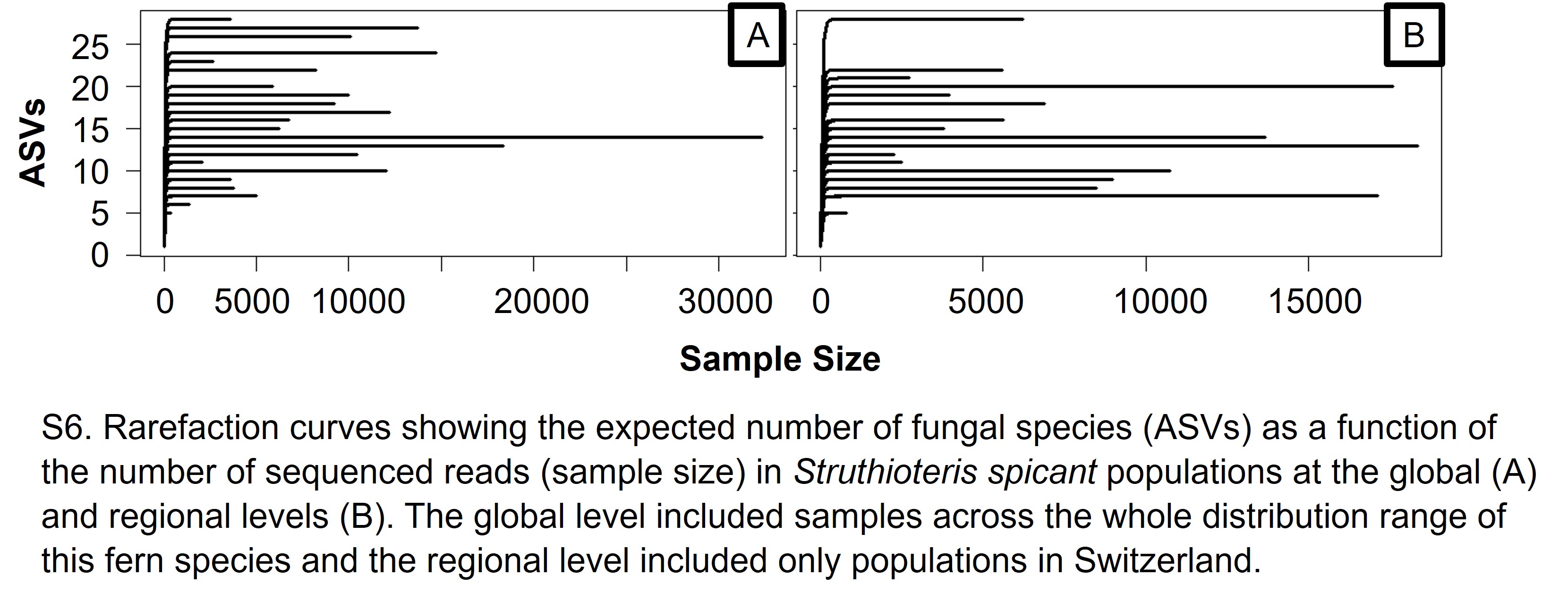

Supplement: Supplementary file 5 [file Image_5.jpeg]
